# Pediatric Medial Condyle of Humerus Fracture Dislocation: A Case Report

**DOI:** 10.7759/cureus.43942

**Published:** 2023-08-22

**Authors:** Paige Matijasich, David Yatsonsky, Tony Dong, Brandon Sharkey, Adrian Lewis

**Affiliations:** 1 Department of Orthopaedic Surgery, The University of Toledo College of Medicine and Life Sciences, Toledo, USA; 2 Department of Pediatric Orthopaedic Surgery, ProMedica Toledo Hospital, Toledo, USA

**Keywords:** radiology, elbow dislocation, anatomy, orthopedics, medial condyle fracture, pediatric fracture

## Abstract

We present the case of a six-year-old male patient, status post fall with elbow dislocation, successfully reduced. At the time of injury, he had normal appearing x-rays, signs of a mildly discrete chip, and possible epicondyle ossification, but no definitive fracture. A medial condyle fracture with dislocation was missed at the original time of injury. The patient returned several years later with mild elbow stiffness and intermittent pain. CT scan at this time demonstrated nonunion of the medial condyle. The patient underwent subsequent open reduction and internal fixation (ORIF) and is currently doing well clinically. Although rare, a medial condyle dislocation fracture needs surgical intervention to reduce morbidity, and therefore, should be in the differential when working up a pediatric fracture.

## Introduction

Fractures from accidental trauma are one of the most common injuries sustained by the pediatric population and are a frequent cause of emergency room visits. The overall risk of a fracture occurring throughout childhood and adolescence is 20-30%, with children between 10-14 years old having the highest incidence [1,2]. Upper extremity fractures account for approximately two-thirds of fractures in the pediatric population [3]. Although fracture sites vary by age and sex, elbow fractures continue to be a common type of pediatric fracture and account for 5-10% of all childhood fractures [1,4]. The most common type of pediatric elbow fracture is the supracondylar fracture, accounting for over 50% of pediatric elbow fractures (50-70%). Lateral condylar (17-34%) and medial epicondylar fractures (10%) are the next two most common [4].

In the case presented in this paper, we will explore a less commonly occurring fracture: medial condylar fracture dislocation. Although this fracture is considered rare, it is important to be able to recognize the signs and symptoms in order to reduce the associated morbidity. This case explores the importance of early recognition of pediatric fractures and discusses possible strategies for diagnosing medial condyle dislocation fractures.

## Case presentation

**Figure 1:**
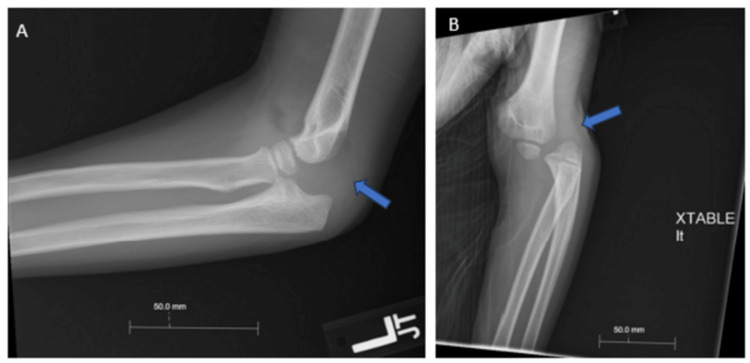
Lateral (A) and anteroposterior (B) x-ray demonstrating dislocation and effusion of the left elbow

Our patient was a six-year-old male who presented with a dislocated left elbow after failed reduction attempts at a level-one trauma centerfacility (Figure 1). The patient underwent successful closed reduction under conscious sedation with ketamine.

**Figure 2:**
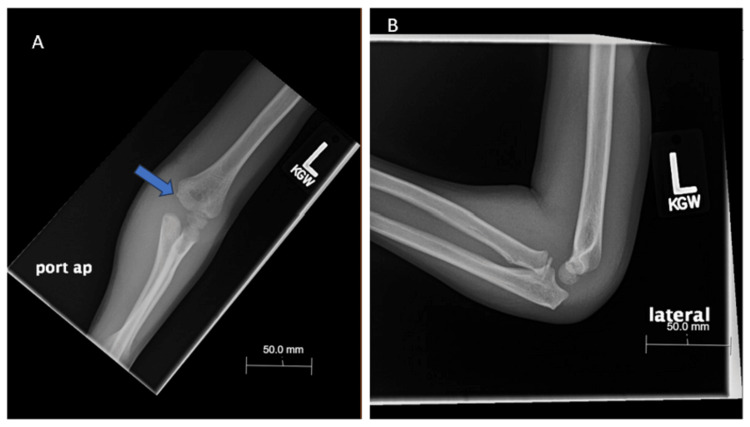
(A) Post-reduction x-ray (anteroposterior) of left elbow demonstrating reduced elbow dislocation, bony irregularity medial humeral condyle; (B) Post-reduction x-ray (lateral) of left elbow demonstrating reduced elbow dislocation

Post-reduction x-rays demonstrated a well-reduced elbow without apparent fracture (Figure 2). The patient was placed into a well-padded posterior slab splint and was discharged with a sling for comfort and clinical follow-up.

**Figure 3:**
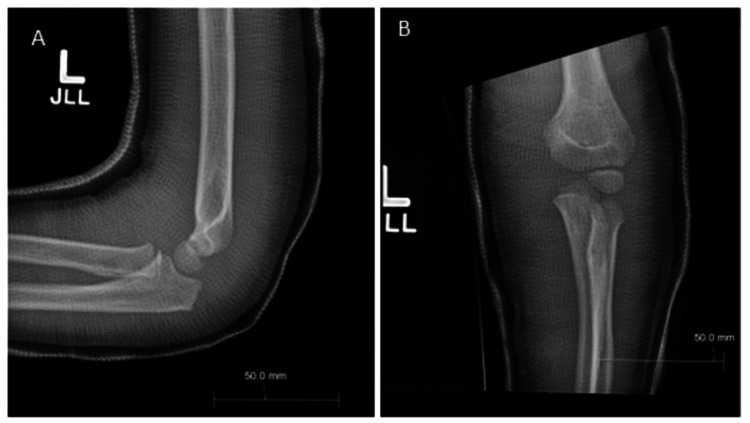
Lateral (A) and anteroposterior (B) radiographs of the left elbow five days post reduction

At the clinical follow-up five days later, the splint was removed, and x-rays demonstrated a well-reduced elbow without periosteal reaction, fracture, and stable effusion (Figure 3).

**Figure 4:**
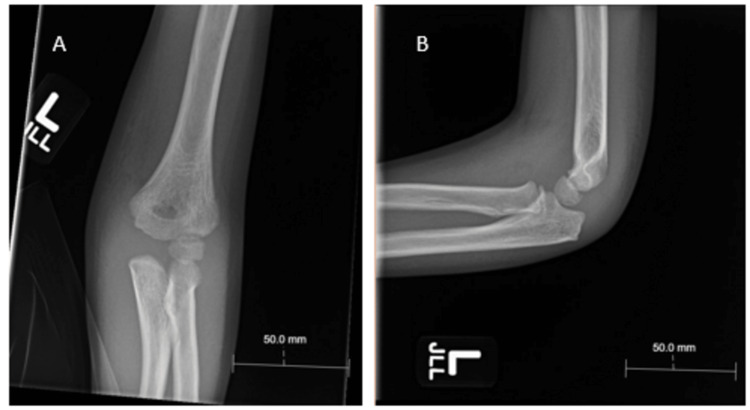
(A) Anteroposterior and (B) lateral x-rays of the left elbow two weeks post injury.

He was placed in a long-arm cast for continued immobilization at that time. At the one-week follow-up, the cast was removed, and X-rays again demonstrated a well-reduced elbow without apparent fracture. There was a very small rim of calcification in the medial soft tissues visualized (Figure 4).

**Figure 5:**
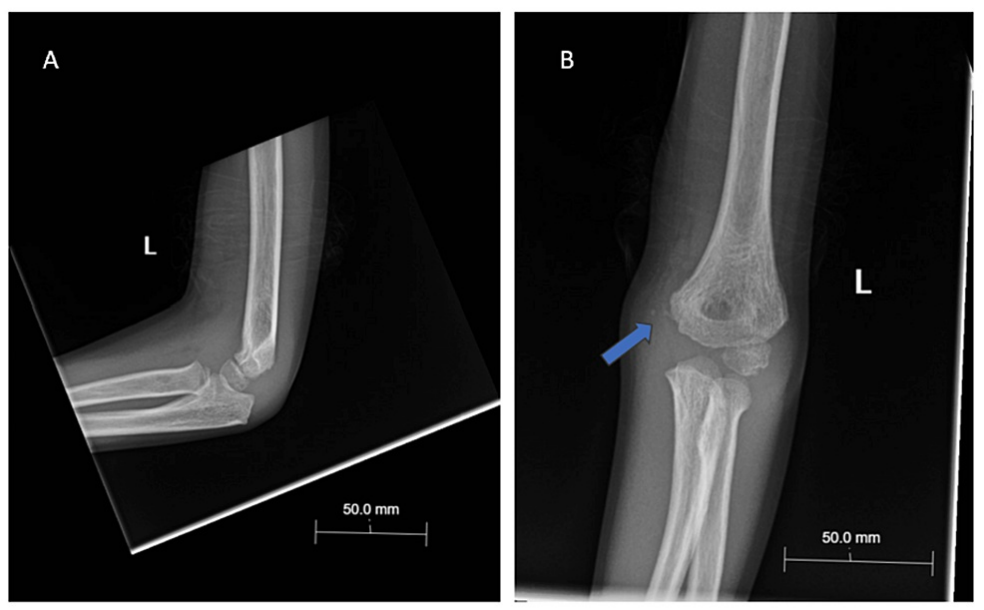
(A) X-ray (lateral view) of the left elbow six weeks post injury; (B) X-ray (anteroposterior) of the left elbow six weeks post injury demonstrating mild calcific osseous abnormality in the medial aspect of the distal humerus

However, this very small rim of calcification was in the expected location of the medial epicondyle ossification center, and the patient's elbow appeared otherwise stable and well-reduced. At six weeks post injury, the patient was returning to normal function, and x-rays continued to show only additional ossification at the location of the medial epicondyle (Figure 5A). There was a small second ossification area that was thought to possibly represent sequelae of multiple reduction attempts resulting in mild heterotopic ossification (Figure 5B). Continued follow-up through six months after injury continued to demonstrate a well-reduced elbow with an improving range of motion (ROM) and continued ossification at the medial aspect of the elbow. Of note, all radiographs through initial follow-up to six months were interpreted as normal.

**Figure 6:**
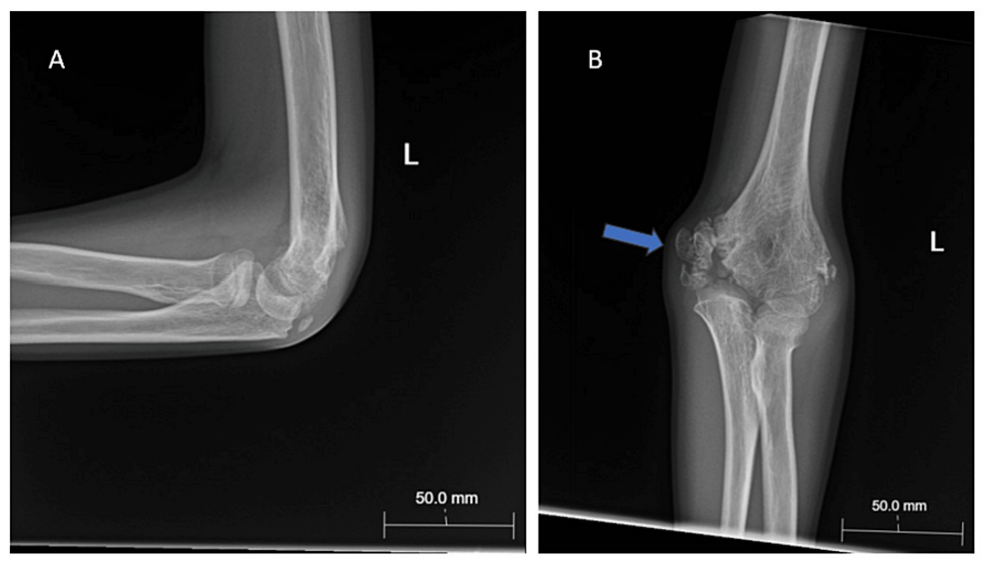
(A) X-ray (lateral view) of left elbow five years post injury; (B) X-ray (anterioposterior view) of left elbow demonstrating chronic nonunion left medial condyle and epicondyle and fragmentation of ossification center

At five years post injury, the patient presented with a new and unrelated metacarpal fracture. During routine follow-up for this injury, he noted approximately six months of intermittent discomfort on the medial elbow with some popping during pushups. Physical exam revealed very mild tenderness along the medial elbow with ROM between 25-130 degrees while in full pronation and while in 45 degrees of supination. X-rays obtained at this visit demonstrated a chronic nonunion of a medial condyle fracture (Figure 6).

**Figure 7:**
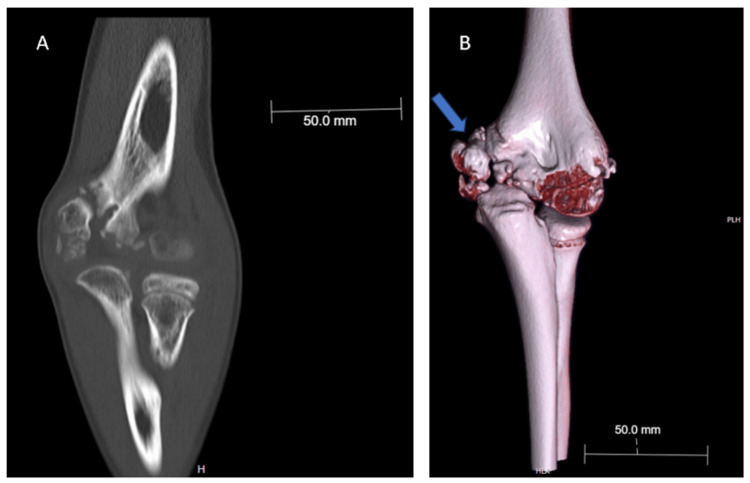
CT scan of the bone (A) and CT three-dimensional reconstruction (B) of the left elbow five years post injury demonstrating chronic nonunion in medial condyle and medial epicondyle with fragmentation of ossification center

A CT scan was done for better evaluation of the bony surfaces as well as evaluation of the joint line. CT confirmed nonunion of the medial condyle with multiple areas of ossification of the medial condyle and epicondyle (Figure 7).

**Figure 8:**
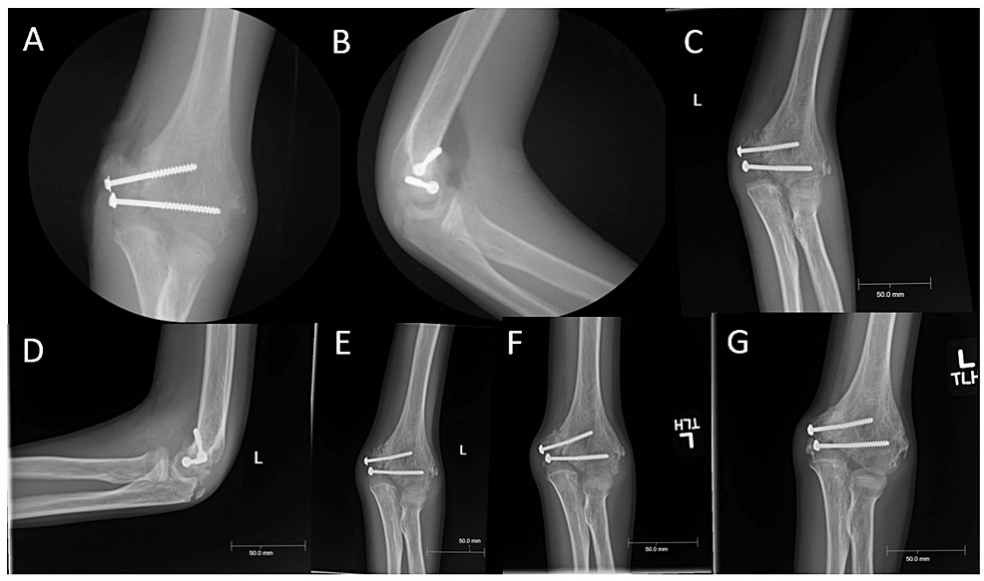
(A) Anteroposterior view of left elbow intraoperatively, demonstrating reduced fracture nonunion; (B) Intraoperative x-ray (lateral view) of left elbow; (C) Anteroposterior view of left elbow two weeks after surgery; (D) Lateral view of left elbow two weeks after surgery; (E) Anterioposterior view of left elbow six weeks after surgery; (F) Anterioposterior view of left elbow 12 weeks after surgery; (G) Anterioposterior view of left elbow six months after surgery

CT demonstrated mild incongruity of the articular surface as well as nonunion of the medial condyle with loose bodies. These findings were discussed with the patient and his family at length as well as operative vs nonoperative options for treatment. Due to intermittent pain and time left for continued development of the elbow, operative stabilization of nonunion was decided upon. The patient underwent open reduction and internal fixation with an autologous bone graft from the bare area on the medial condyle. He was immobilized for two weeks postoperatively and then began with formal physiotherapy to improve ROM. He also received a dynamic extension splint from occupational therapy (OT). At six months post this surgery, ROM was satisfactory to the patient and his family, and there was an improvement when playing sports. ROM at that time was 20-130 degrees, and he was almost able to get to full supination. X-rays through the postoperative course demonstrate the healing of nonunion sites with the maintenance of orthopedic implants (Figure 8).

## Discussion

Pediatric medial condyle fractures of the humerus (MCFH) are rare and account for less than 1-2% of elbow injuries and 4% of distal humeral epiphyseal injuries [5,6]. Due to the rarity of the fracture, diagnosis can be challenging and often leads to either misdiagnosis or delayed diagnosis. In one retrospective study of 21 medial condylar fractures, the stated complication rate for these fractures was upwards of 33%, so early intervention is integral [5]. Because medial condylar fractures are Salter-Harris type IV injuries with intra-articular damage, these fractures are prone to nonunion and likely require surgical consultation.

In 1965, Kilfoyle created a classification system to better understand the various types of injury and treatment methods surrounding medial condyle fractures of the humerus [7]. A type 1 fracture is characterized by a fracture line that extends down to the physis. Type 2 fractures consist of fracture lines that extend through the physis, which remains nondisplaced. The type 3 classification is characterized by a fracture line that extends through the physis and the fragment is displaced and rotated [7].

With MCFH, there is a clear association between delays in diagnosis and poor outcomes [6,8-10]. Medial condyle fractures have characteristics of Salter-Harris IV physeal fractures because they commonly extend from the metaphysis through the physis and into the epiphysis [8,9,11]. In medial condyle fractures, there is also likely epiphyseal plate injury, so inappropriate initial management increases the risk of altered joint mechanics, growth arrest, malalignment, and lifelong functional impairment for the patient [12]. This supports the need for prompt diagnosis and treatment of these fractures.

Although the exact percentage varies, studies have suggested that the overall risk of sustaining a fracture in childhood is about 10-25%, with the lifetime risk of sustaining a fracture being 27% and 42% for females and males, respectively [2]. Of these fractures, upper extremity fractures are more common [3]. Medial condyle fractures, specifically, are not common fractures, accounting for only about 1-2% of pediatric fractures [5-10]. However, if missed, these fractures can lead to lifelong morbidity. Medial condyle fractures may be difficult to diagnose in younger children with the fracture mistaken for a simple avulsion of the medial epicondyle or may even be completely missed. The likelihood of missing medial condyle fractures is most likely due to anatomy and ossification changes in the pediatric population. The chronological order of elbow ossification center appearance is as follows: the capitellum first, followed by the radial head, medial epicondyle, olecranon, trochlea, and last, the lateral epicondyle [13]. The ability to diagnose medial condylar fractures tends to align with the amount of ossification of the trochlea and medial condyle. The smaller the ossified part, the harder it is to recognize a medial condyle fracture [7-9]. The medial epicondyle and trochlea tend to ossify by the age of nine years for females and by the age of 11 for males [13,14]. The trochlea may not be completely ossified until 13 years of age. If ossification has not started or is not completed, diagnosis of fracture is much more difficult. The most important ossification center responsible for the misdiagnosis of a medial condyle fracture is cited to be the trochlear ossification center due to its proximity to the medial condyle and its ability to obscure visualization radiographically [11]. Multiple studies have stated the risk of misdiagnosing or missing this fracture is highest in children younger than six years, which is likely due to the unossified trochlear center [7-9,11]. 

When it comes to diagnosing medial condyle fractures, one study suggests looking for the c-sign on X-ray, which is a sickle-shaped, malrotated small metaphyseal bone scale with c-formation, which is also described in multiple other case reports [9,11,15]. They consider this c-sign to be pathognomonic and if present on the medial side of the elbow, medial condyle fracture should be considered and/or excluded [11]. Two different studies suggest any dislocation of the medial epicondyle in a child whose trochlear nucleus of ossification has not appeared yet should arouse suspicion for a medial condyle fracture [9,16]. A study by Zukotynski et al. suggests that severe edema, ecchymosis, and tenderness to palpation on the medial side of the elbow in the presence of an apparent avulsion fracture in patients younger than six years old could suggest the possibility of a displaced fracture of the medial humeral condyle [16]. Therefore, additional assessment including up to four views radiographically, evaluation under anesthesia, arthrography, or advanced imaging should be warranted [17]. Other studies list subtle radiographic clues suggestive of medial condyle fracture including the presence of an apparent “fleck of bone” over the medial side of the elbow, a fat pad sign, or an especially widely displaced medial epicondyle fragment [18-20]. Research currently available suggests that plain radiographs may not be enough to diagnose medial condyle fractures, especially in younger children. Therefore, studies have suggested having a high index of suspicion for a medial condyle fracture in a pediatric patient who presents with medial elbow pain, decreased ROM, swelling, redness, or bruising, especially in the setting of a concomitant dislocation of the elbow [17]. If a pediatric patient presents with these signs and symptoms, a greater workup should be done to prevent possible long-term ramifications if this injury is not identified and treated promptly. 

## Conclusions

This case report outlines a delayed and misdiagnosed medial condyle of the humerus fracture and discusses the need for prompt diagnosis and treatment of a commonly missed pediatric fracture. It is easy to miss medial condyle fractures and even mistake them for a simple avulsion. The late diagnosis of a medial condyle fracture can disrupt the growth of important elbow components in children leading to significant morbidity later in life. With early diagnosis, accurate reduction and internal fixation can be done to promptly treat and prevent chronic nonunion of the medical condyle fracture.

There are multiple techniques to attempt to fix the nonunion if discovered late. In the current case, an open reduction and internal fixation with an autologous bone graft from the bare area on the medial condyle of the humerus was done. Our method seems to have been quite successful. This case report also highlights the signs and symptoms that should raise suspicion about these fractures and discusses the importance of further imaging in order to diagnose and prevent lifelong morbidity. The literature on this topic is limited due to the rarity of medial condyle fractures, and this case offers expanded insight into the risks, diagnosis, and treatment of these fractures, encouraging medical providers to be aware of the possibility of this fracture, especially in the pediatric population. 
